# A Case of Alveolar Abscess

**Published:** 1859-11

**Authors:** 


### A CASE OF ALVEOLAR ABSCESS.

About the first of last December, I was called on to fill a tooth —an upper canine—which was affected with abscess, of several months' standing. I cleansed out the cavity of decay, and the canal, and passed a broach drill through the apex of the fang. Then, with a quill broach, armed with cotton, I pumped creosote up the canal, till I saw, by its cauterizing effects, that it had passed entirely through the fistulous opening. I filled the tooth immediately, leaving the canal unfilled.

I saw the tooth a few days ago. Every thing about it is, and has been satisfactory.

I suppose it would be impracticable, in most cases, to prepare the tooth so thoroughly at a single sitting. But patience and *mild* means will mostly succeed. W.

